# Should vitamin B_12_ status be considered in assessing risk of neural tube defects?

**DOI:** 10.1111/nyas.13574

**Published:** 2018-01-29

**Authors:** Anne M. Molloy

**Affiliations:** ^1^ School of Medicine and School of Biochemistry and Immunology, Trinity College Dublin Trinity Biomedical Sciences Institute Dublin Ireland

**Keywords:** vitamin B_12_, cobalamin, folic acid, folate, maternal blood

## Abstract

There is a strong biological premise for including vitamin B_12_ with folic acid in strategies to prevent neural tube defects (NTDs), due to the closely interlinked metabolism of these two vitamins. For example, reduction of B_12_ deficiency among women of reproductive age could enhance the capacity of folic acid to prevent NTDs by optimizing the cellular uptake and utilization of natural folate cofactors. Vitamin B_12_ might also have an independent role in NTD prevention, such that adding it in fortification programs might be more effective than fortifying with folic acid alone. Globally, there is ample evidence of widespread vitamin B_12_ deficiency in low‐ and middle‐income countries, but there is also considerable divergence of vitamin B_12_ status across regions, likely due to genetic as well as nutritional factors. Here, I consider the evidence that low vitamin B_12_ status may be an independent factor associated with risk of NTDs, and whether a fortification strategy to improve B_12_ status would help reduce the prevalence of NTDs. I seek to identify knowledge gaps in this respect and specify research goals that would address these gaps.

## Introduction

Evidence that folic acid can prevent neural tube defects (NTDs) is based on consistent results from well‐conducted randomized trials, backed up by evidence that food fortification with folic acid can lower the prevalence of NTDs at the population level.^1^ In sharp contrast to this, the available evidence relating to its metabolic partner vitamin B_12_ is mainly confined to observational data on blood concentrations of the vitamin in NTD‐affected women, partially because vitamin B_12_ was never assessed as a factor in early studies of the topic. This is unfortunate considering current knowledge of the extent of B_12_ deficiency worldwide and the likelihood that B_12_ may play a role in NTD prevention along with folic acid.

### Evidence supporting biological plausibility on a link between vitamin B_12_ status and risk of NTDs

Vitamin B_12_ is an enzymic cofactor for only two biochemical reactions in humans. One of these enzymes (methylmalonylCoA mutase) is involved in catabolism of some fats and amino acids and is not linked to folate metabolism. The metabolic interaction between folate and vitamin B_12_ is positioned at the other B_12_‐dependent enzyme—methionine synthase—which converts homocysteine to methionine using 5‐methyl tetrahydrofolate (methyl folate) as the methyl donor.^2^ The continuous recycling of homocysteine to methionine through this enzyme is an essential cellular reaction wherein folate‐derived methyl groups are transferred to a multitude of products, including methyl‐DNA and methylated proteins that contribute to gene expression and gene silencing mechanisms (i.e., epigenetic control of cellular functions). One current hypothesis for the role of folate in neural tube formation relates to the importance of such epigenetic processes in orchestrating the flow of molecular processes that must be achieved during neural tube closure.^3^, ^4^ Vitamin B_12_‐dependent methionine synthase is also the checkpoint reaction through which folate molecules are incorporated into cells, because folate in the circulation exists predominantly as methyl folate. Once within the cell, having released the methyl group to homocysteine through the methionine synthase reaction, folate cofactors then can function in the transfer of other one‐carbon groups (e.g., formyl‐ or methylene‐) to nucleotide molecules destined for synthesis of DNA (i.e., cell proliferation) or to other molecular products.

The other main hypothesis relating folates to neural tube formation is based on the requirement for folate in DNA synthesis and cell proliferation.^5^ A portion of the formyl‐ or methylene folate derivatives is also converted to the methyl‐form, which is channeled through the methionine synthase reaction for methionine regeneration, as noted above. The methyl folate trap hypothesis was proposed in 1972 to explain the similarity of some clinical features of folate and vitamin B_12_ deficiencies.^6^ In this scenario, impaired methionine synthase results in a cellular accumulation of the substrate methyl folate, which does not have an alternative metabolic outlet. The cell therefore becomes functionally deficient in folates, and folate‐dependent DNA synthesis is compromised, leading to the classic megaloblastic anemia that characterizes folate and B_12_ deficiency. It is important to note that folic acid can bypass vitamin B_12_‐dependent methionine synthase to enter the cellular folate pool and resume folate functions in relation to DNA synthesis, but it cannot contribute to the provision of methyl groups for methylation functions described above. An awareness of this intertwined biochemistry of folate and vitamin B_12_ underlies the strong biological premise for considering vitamin B_12_ as well as folic acid in strategies for NTD prevention.

### Historical perspective in relation to vitamin B_12_ and NTD prevention studies

The studies on vitamin deficiencies and NTDs conducted by Smithells and coworkers in the 1970s were relatively inclusive within the framework of the type of micronutrient deficiency that was expected to be present in young women in nutritionally deprived social groups. Concentrations of serum vitamins A, riboflavin, B_6_, and folate, plus red cell folate (RCF) and white blood cell ascorbic acid, were measured in the blood of NTD‐affected women in the study by Smithells *et al*. in 1976.^7^ Although folate deficiency was a top candidate for causality, Smithells always speculated that one or more vitamins could also be involved. The preparation (Pregnavite Forte F) that he used in his follow‐up intervention trials to test the hypothesis that preconceptional vitamin supplementation would prevent NTDs contained eight vitamins and was taken three times a day to deliver a daily dose of vitamin A (4000 IU), vitamin D_2_ (400 IU), vitamin C (40 mg), and the B vitamins thiamine (1.5 mg), riboflavin (1.5 mg), nicotinamide (15 mg), pyridoxamine (1 mg), folic acid (0.36 mg), ferrous sulfate (252 mg), and calcium phosphate (480 mg).^8^ The results showed a highly protective effect of treatment, with an approximate sevenfold reduction in recurrence rate among fully treated women.^8^ For completeness, his group also measured changes in maternal status of the vitamins after intervention and demonstrated substantial improvements in the blood vitamin profiles.^9^ For example, they observed a doubling of RCF (from 566 to 1083 nmol/L) and a tripling of serum folate (from 19 to 59 nmol/L) after intervention. The subsequent Medical Research Council (MRC) randomized controlled trial included a similar cocktail of vitamins (B_1_, B_2_, B_3_, B_6_, A, C, and D plus folic acid).^10^ While vitamin B_12_ was not included for the reasons given below, it is worth noting that the MRC trial did include the other B vitamins that are involved in folate‐dependent one‐carbon metabolism: B_6_ (pyridoxal), B_2_ (riboflavin), and B_3_ (as nicotinamide). The multivitamin combination without folic acid had no protective effect (relative risk 0.80; 95% confidence interval (CI) 0.32–1.72); ruling out these vitamins as important factors. This was an important guide to future research directions.

The link between folate and vitamin B_12_ was understood at the time of Smithells’ work in the mid‐1970s. However, vitamin B_12_ deficiency was thought to be confined mainly to persons with pernicious anemia—a condition that was known to be largely prevalent in older persons and extremely rare in women of reproductive age. Indeed, several reports documenting case series suggested that pernicious anemia was incompatible with pregnancy.^11^ Pernicious anemia remains a rare and serious disease that is not particularly relevant to the occurrence of NTDs. However, it is now known that vitamin B_12_ deficiency (usually defined as serum total vitamin B_12_ < 148 pmol/L) is a problem of global scale, affecting all populations studied. It is mainly due to inadequate intake or intestinal parasitic infections and reportedly present in up to 70% of adolescents and women of reproductive age in low‐income and predominantly vegetarian countries.^12^ This means that millions of women enter pregnancy with inadequate vitamin B_12_ tissue stores to allow for optimal folate functionality. Pregnancy‐related changes in serum total vitamin B_12_ concentrations occur (and are discussed later), but the critical point is that the neural tube closes in the first 21–28 days of pregnancy, therefore the nonpregnant vitamin B_12_ status that exists immediately before a woman enters pregnancy is the key factor in relation to NTDs.

## What evidence exists for an association of B_12_ with NTDs?

The first suggestion in the literature of an association between NTDs and vitamin B_12_ was a short letter in *Lancet* by Schorah *et al*. in 1980.^13^ The women described by Schorah *et al*. were a subset of those studied by Smithells *et al*.,^7^ who reported lower RCF concentrations in women who were pregnant with an NTD‐affected fetus compared with nonaffected controls. In a follow‐up study, Schorah *et al*. reported that of six NTD mothers and 48 control mothers who had serum total vitamin B_12_ concentrations measured in early pregnancy, three mothers of anencephalic infants had the lowest vitamin B_12_ concentrations (all less than 185 pmol/L compared with a mean (95% range) of 308 (166–5460) pmol/L in pregnant controls).^13^ The authors proposed that a more marked vitamin depletion might be associated with more severe NTDs. This study was too small to form any opinion on the relevance of low vitamin B_12_ in relation to NTDs, and at the time Smithells was well into his intervention study with Pregnavite Forte supplements that did not contain vitamin B_12_ but that demonstrated an approximate sevenfold reduction in NTD recurrence rates compared with women who did not take these supplements.^8^

### Case–control studies

In the following two decades, more than 20 studies addressed the question of whether vitamin B_12_ status might be of relevance to NTD prevention (Table 1). In some of these studies, blood was taken postpartum^14^, ^15^ or at a time for case mothers that was remote from the affected pregnancy.^16^, ^17^, ^18^, ^19^, ^20^, ^21^, ^22^ This was not unreasonable in the context of the leading hypothesis to explain the cause of NTDs and the efficacy of folic acid: that an underlying genetic susceptibility in mothers, fathers, or the developing fetus, exacerbated by low nutritional status, acts to influence folate‐sensitive metabolic processes at the time of neural tube closure. Such a genetic susceptibility might be observed in altered vitamin status in blood or altered plasma total homocysteine (a sensitive biomarker of both folate and vitamin B_12_ status), at any time in an individual's life. Indeed, because of its known toxicity and reactivity, an elevation in homocysteine at the neural crest was an early hypothesis proposed to explain the failure of neural tube closure, and both folate and vitamin B_12_ were measured as factors that, if inadequate, might cause elevation of homocysteine.^23^

**Table 1:** Studies that examined maternal serum or plasma vitamin B_12_ status in relation to neural tube defects

Study	Country	Sample	Time of sampling	Cases/controls (*n*)	Cases/controls concentrations pmol/L	Significant difference: *P* value (if given)
Nasri *et al*.^38^	Tunisia	Serum B_12_	2nd–3rd trimester	75/75	218/264 (median)	*P* = 0.009
Molloy *et al*.^35^ (three cohorts)	(1) Ireland (1983–1984)	Serum B_12_	15 weeks median	95/265	155/179 (median)	*P* = 0.003
	(2) Ireland (1986–1990)	Plasma B_12_	15 weeks median	76/222	180/221 (median)	*P* = 0.024
	(3) Ireland (1986–1990)	Plasma B_12_	15 weeks median	107/414	199/232 (median)	*P* = 0.003
Zhang *et al*.^36^	China (Shanxi) (2004–2005)	Serum B_12_	20 weeks median	84/110	73/91 (geometric mean)	*P* < 0.01
Ray *et al*.^37^	Canada (1993–2004)	Serum holo‐transcobalamin	15–20 weeks	89/422	68/81 (geometric mean)	Yes; OR 2.9 (95% CI: 1.2–6.9)
Gaber *et al*.^16^	Egypt	Serum B_12_	Not pregnant	27/25	207/258 (median)	*P* = 0.025
Gaber *et al*.^16^	Egypt	Serum B_12_	Second trimester	9/10	185/240 (median	*P* = 0.022
Suarez *et al*.^14^	Texas–Mexico border (1995–2000)	Serum B_12_	Postpartum	225/378	317/367 (median)	*P* = 0.001
Afman *et al*.^19^	Netherlands	Plasma B_12_	Not pregnant	46/73	220/220 (median)	No
Afman *et al*.^19^	Netherlands	Plasma holo‐transcobalamin	Not pregnant	46/73	41/50 (median)	No
Wilson *et al*.^21^	Canada (pre‐1998)	Serum B_12_	Not pregnant	58/89	298/350 (mean)	*P* = 0.05
Wright *et al*.^15^	Northern Ireland	Serum B_12_	Postpartum	15/15	148/218 (mean)	*P* = 0.004
van der Put *et al*.^22^	Netherlands	Serum B_12_	Not pregnant (includes men)	60/94	245/255 (median)	No
Wald *et al*.^25^	UK MRC Trial (International)	Serum B_12_	12 weeks	18/75	170/177 (mean)	*P* = 0.05
Adams *et al*.^27^	USA	Serum B_12_	Second trimester	33/132	297/319	No
Steegers‐Theunissen *et al*.^17^	Netherlands	Serum B_12_	Not pregnant	41/50	198/195 (mean)	No
Steegers‐Theunissen *et al*.^26^	Netherlands	Serum B_12_	Second trimester	27/31	219/238 (mean)	No
Wild *et al*.^20^	UK	Serum B_12_	Not pregnant	29/29	331/361 (median)	No
Mills *et al*.^34^	Finland (1983–1989)	Serum B_12_	6–16 weeks	78/150	356/384 (mean)	No: OR 1.05 (95% CI: 0.9–1.2)
Economides *et al*.^33^	Oxford, UK	Serum B_12_	14–21 weeks	8/24	151/170 (median)	No
Yates *et al*.^18^	Scotland, UK (1980s)	Serum B_12_	Not pregnant	20/20	221/236 (mean)	No
Molloy *et al*.^24^	Ireland (1980–1982)	Serum B_12_	15 weeks median	28/363	219/204 (median)	No
Schorah *et al*.^13^	UK (1970–1972)	Serum B_12_	<13 weeks	6/48	213/308 (mean)	No

Most of the earlier studies included relatively few case mothers and were probably underpowered to detect the small differences in serum vitamin B_12_ or in plasma total homocysteine that might have been expected (Table 1).^18^, ^19^, ^20^, ^24^, ^25^, ^26^, ^27^ However, several studies reported elevated homocysteine in plasma and amniotic fluid.^23^, ^26^, ^28^ It is of interest that six studies examined concentrations of vitamin B_12_ or its binding proteins in amniotic fluid, all around 20 weeks of gestation (Table 2). The numbers were small, but nevertheless five reported significantly lower concentrations of total vitamin B_12_ or vitamin B_12_ bound to its transport protein transcobalamin (TCII) in pregnancies affected by NTDs.^26^, ^29^, ^30^, ^31^, ^32^, ^33^ In some of these studies, vitamin B_12_ in serum was not significantly changed. Although these reports provided intriguing new data on the role of vitamin B_12_ in relation to NTDs, there is a high risk of confounding because of poor case–control matching. Publication bias is also extremely likely in the studies reported over this period given the lack of negative reports despite low power to detect effects.

**Table 2:** Studies that examined maternal amniotic fluid vitamin B_12_ status in relation to neural tube defects

Study	Country	Time of sampling	Cases/controls (*n*)	Cases/controls concentrations pmol/L	Significant difference: *P* value (if given)
Dawson *et al*.^30^	USA (>30% Hispanic population)	15–20 weeks	11/29	226/618 (mean)	*P* < 0.001
Steen *et al*.^31^	Georgia, USA	14–18 weeks	16/64	111/399 (mean)	*P* = 0.02
Steegers‐Theunissen *et al*.^26^	Netherlands	Second trimester	27/31	481/379 (mean)	No
Weekes *et al*.^32^	Alabama, USA	14–22 weeks	8/47	140/600 (mean)	*P* < 0.001
Economides *et al*.^33^	UK	14–21 weeks	8/24	92/207 (median)	*P* < 0.01
Gardiki‐Kouidou and Seller^29^	UK (1982–1987)	15–22 weeks	26/65	110/162 (median)	*P* < 0.04

The best evidence for an involvement of low vitamin B_12_ status comes from the large population‐based studies (>80 cases) that could assess separate and combined risk with low blood folate status. There are six such publications, and, importantly, they cover a range of ethnicities, including Irish, Chinese, predominately Hispanic (Mexico–Texas border), Canadian (mixed population), Tunisian, and Finnish. All of them, apart from the study in Finland, found positive associations, in that they linked low maternal serum vitamin B_12_ status to increased risk of NTDs, but it may be relevant that the negative study was conducted in a population in which the average serum B_12_ concentration of participants was higher than in any of the other studies noted in Table 1.^34^

The studies that showed a significant association between vitamin B_12_ status and NTD risk were carried out (1) on three Irish early‐pregnancy populations before voluntary fortification of foods was common in Ireland;^35^ (2) on a middle‐to‐late pregnancy population of high‐risk Chinese women in the Luliang mountain region of Shanzi provence;^36^ (3) on a postpartum population of women living on the Texas–Mexico border during the introduction of folic acid fortification in the United States;^14^ (4) on a postfortification early‐pregnancy population of women in Canada,^37^ and (5) on a population of women attending a national maternity and neonatology center in Tunis that represents all NTD cases observed throughout Tunisia.^38^ All studies observed significantly lower maternal blood concentrations of vitamin B_12_ or holotranscobalamin (holoTC; the fraction of vitamin B_12_ that is transported in the blood circulation on TCII and is destined for tissue uptake). All studies reported an approximate tripling (or greater) of risk between the lowest and highest quartile or quintile of serum vitamin B_12_ or quartile of holoTC (Table 3). Two recent meta‐analyses were carried out on existing data. Both concluded that maternal B_12_ deficiency is an important consideration in relation to NTDs, although it must be said that discrepancies and lack of clarity between the data included in the meta‐analyses and data reported in the original studies indicate that these meta‐analyses were limited in scope. The derived estimates should be interpreted with caution.^39^, ^40^

**Table 3:** Odds ratios for effect of low vitamin B_12_ status on maternal risk of having an NTD‐affected pregnancy

Study	Quantile of distribution in controls					
(sample timing)		1	2	3	4	5
Suarez *et al*.^14^	B_12_ (pmol/L)	92*–*275	276*–*324	325*–*404	405*–*484	485*–*2021
(5*–*6 weeks postpartum)	Crude OR [95% CI]	3.0 [1.4*–*6.3]	1.6 [0.7*–*3.6]	1.7 [0.8*–*3.8]	1.1 [0.5*–*2.6]	Referent
	Case/control (*n*)	55/37	30/37	32/37	21/37	19/38
Ray *et al*.^37^	holoTC (pmol/L)	≤55.3	>55.3*–*84	>84*–*121	>121	
(15*–*20 weeks gestation)	Crude OR [95% CI]	2.0 [1.1*–*3.9]	1.1 [0.6*–*2.3]	1.0 [0.5*–*2.1]	Referent	
	aAdjusted OR [95% CI]	2.9 [1.2*–*6.9]	2.0 [0.8*–*5.1]	1.1 [0.4*–*2.9]	Referent	
	Case/control (*n*)	35/106	19/105	18/106	17/105	
Zhang *et al*.^36^	B_12_ (pmol/L)	<55	≥55			
(5*–*41 weeks gestation)	Crude OR [95% CI]	3.7 [1.6*–*8.7]	Referent			
	bAdjusted OR [95% CI]	5.0 [1.9*–*12.7]	Referent			
	Case/control (*n*)	21/9	63/101			
Molloy *et al*.^35^	B_12_ (pmol/L)	<140	140*–*178	179*–*221	>221	
Cohort 1 (NTD pregnancy)	Crude OR [95% CI]	3.2 [1.5*–*6.8]	2.8 [1.3*–*6.0]	1.8 [0.8*–*4.1	Referent	
(11*–*20 weeks gestation)	cAdjusted OR [95% CI]	3.1 [1.5*–*6.7]	2.6 [1.4*–*5.7]	1.8 [0.8*–*4.1]	Referent	
	Case/control (*n*)	36/67	29/66	19/66	11/66	
Molloy *et al*.^35^	B_12_ (pmol/L)	<186	187*–*232	233*–*298	>298	
Cohort 2 (other pregnancy)	Crude OR [95% CI]	2.9 [1.5*–*5.5]	1.4 [0.7*–*2.9]	1.9 [1.0*–*3.7]	Referent	
(11*–*21 weeks gestation)	dAdjusted OR [95% CI]	2.8 [1.4*–*5.3]	1.3 [0.7*–*2.8]	1.8 [0.9*–*3.6]	Referent	
	Case/control (*n*)	43/103	21/104	28/104	15/103	
Molloy *et al*.^35^	B_12_ (pmol/L)	<175	175*–*221	222*–*270	>270	
Cohort 3 (NTD pregnancy)	Crude OR [95% CI]	3.0 [1.4*–*6.4]	1.5 [0.7*–*3.4]	1.2 [0.5*–*2.8]	Referent	
(12*–*20 weeks gestation)	dAdjusted OR [95% CI]	2.5 [1.1*–*5.3]	1.6 [0.7*–*3.6]	1.1 [0.5*–*2.7]	Referent	
	Case/control (*n*)	34/55	18/57	12/55	12/55	
Nasri *et al*.^38^	B_12_ (pmol/L)	33*–*230	231*–*316	317*–*575		
(2nd–3rd trimester)	Crude OR [95% CI]	2.6 [1.2*–*5.7]	1.1 [0.5*–*2.7]	Referent		
	Case/control (*n*)	41/25	18/25	16/25		

a Adjusted for serum folate, anthropometric, and demographic factors.

b Adjusted for anthropometric/demographic factors.

c Adjusted for serum folate.

d Adjusted for red cell folate.

### Observations from clinical trials

From the above studies, it seems reasonable to assume, albeit based on observational blood data (low‐grade evidence), that inadequate maternal vitamin B_12_ status is a risk factor for having an NTD‐affected pregnancy. Two additional anecdotal comments support this assumption. First, in contrast to the MRC randomized trial noted earlier (which reported six NTD recurrences among 593 women who received folic acid and 21 NTDs among 602 women who did not receive folic acid), vitamin B_12_ was included in the daily supplement for the contemporary Hungarian randomized clinical trial that found a protective effect of multivitamins against first occurrence of NTDs.^41^ This latter trial (in which women received supplements containing 12 vitamins, including 0.8 mg of folic acid and 4 μg vitamin B_12_) appeared to show 100% protection (zero occurrences among 2104 women who received multivitamins compared with six occurrences among 2052 women who received the trace‐element placebo). The underlying NTD rate in the Hungarian study population was approximately two per thousand at the time, so finding no NTDs in over 2000 pregnancies seemed to show a greater protective effect than would have been expected, assuming a 70% protective effect for folic acid alone, as recorded in the MRC trial^10^, ^41^ (although zero occurrences would have been within the CI of a true 70% effect). Therefore, while the result could be due to chance, it is also possible that a portion of the risk reduction was due to an independent or synergistic effect of vitamin B_12_ (see further discussion below).

### Observations from genetic studies

Second, there is a potential corroboration of the above evidence in studies of genetic factors that might be associated with increased risk of NTDs. To date, the search for genetic polymorphisms that might contribute to the risk of NTDs has had little success in identifying singularly important genes or polymorphisms that confer a major portion of the genetic risk for these birth defects.^5^, ^42^ The best‐described genetic variant in folate metabolism is a single‐nucleotide polymorphism within the gene *MTHFR* (rs1801133, c.677C>T), which is a moderate maternal risk factor for having an NTD‐affected pregnancy in some populations,^43^, ^44^ but apparently not in others.^45^ Genes involved in vitamin B_12_ metabolism, transport, and absorption have also been studied,^45^, ^46^, ^47^, ^48^, ^49^, ^50^, ^51^ and it seems that common genetic variation in genes involved in trafficking of vitamin B_12_ may be associated with increased maternal risk of having an NTD‐affected pregnancy.^51^, ^52^, ^53^ Possibly, the most interesting of these is one report of a strong association between risk of NTD and polymorphisms in the TCII receptor gene (TCblR; CD320) that facilitates holoTC uptake into tissues.^53^ In this study, the most significant risk factors were two tightly linked rare variants (rs2336573 and rs9426) that were highly significant in a recessive model and were not detected in nearly 1000 controls but were present in eight and seven cases with spina bifida, respectively.^53^ The variants conferred an approximate sixfold risk to cases;^53^ however, because of low frequency, it must be said that they are unlikely to contribute greatly to the general risk of NTD in the population. Nevertheless, the study helps to establish the importance of vitamin B_12_ as a relevant vitamin, and the data are consistent with the notion of impaired vitamin B_12_ metabolism being a risk factor that might be overcome by folic acid supplementation, depending on whether the primary event that is disrupted during neural tube formation relates to the provision of formyl‐ or methenyl‐groups for DNA synthesis or the provision of methyl‐groups for epigenetic signaling events or other methylation reactions (as outlined in the introductory paragraph).

## Is the reported effect of vitamin B_12_ independent of folate?

It has been considered that low levels of vitamin B_12_ may reflect low levels of folate; however, analysis of both vitamins in the large studies noted above suggests that they are independent risk factors. For example, the studies carried out in the United States during the introduction of folic acid fortification^14^ and postfortification in Canada^37^ found no increased risk by serum folate status, which was relatively high in both studies (serum folate above 25 nmol/L for cases and controls in the U.S. study and above 30 nmol/L for cases and controls in the Canadian study). In the Canadian study, the researchers calculated an odds ratio of 2.9 (95% CI 1.2–6.9) for serum holoTC in the lowest versus highest quartile in NTD‐affected mothers, after adjusting for serum folate and other relevant confounders. They suggested that, in postfortification Canada, about 34% of NTDs could be due to low maternal vitamin B_12_ status.^37^

Further evidence of an independent role for vitamin B_12_ comes from the studies carried out in Ireland and China on high‐risk populations before the implementation of folic acid fortification. In a nested case–control study of folate and vitamin B_12_ status in early‐pregnancy samples (average 15 weeks of gestation) from 81 Irish women with an NTD‐affected pregnancy and 247 pregnant controls, Kirke *et al*. found no interaction between plasma folate and plasma vitamin B_12_.^54^ Moreover, when assessed simultaneously, having separated risk groups by quartile of folate or vitamin B_12_ concentration using control data as the reference cutoffs, the risk increased with decreasing B_12_ at each quartile of plasma folate, and the highest odds ratio of an NTD‐affected birth was for mothers in the bottom quartile of both plasma folate and plasma vitamin B_12_ (5.4 (95% CI 1.2–25.2) using mothers in the top quartile for both vitamins as the reference).^54^ Zhang *et al*.^36^ reported results from 89 pregnant case mothers and 122 controls that agree closely with the Irish study above,^54^ and they also highlight the independent nature of the association of NTDs with low vitamin B_12_ status.

A subset of the Kirke *et al*. cohort (76 case and 222 control mothers in whom supplement use could be excluded) was included in the later Irish study that specifically examined serum vitamin B_12_ status in relation to the risk of NTDs in three independent cohorts.^35^ In that study, two of the independent cohorts consisted of women currently undergoing an NTD‐affected pregnancy. The third cohort included women who had previously experienced an NTD‐affected pregnancy, but the sample on this occasion was taken during an unaffected pregnancy. These women also had significantly lower B_12_ status compared with matched controls, but their median RCF, while lower, was not significantly different (578 versus 659 nmol/L; *P* = 0.079).^35^ It is difficult to interpret the lower B_12_ status in these women, since the pregnancies did not result in NTDs, but one possibility is that an underlying long‐term deficiency or inadequacy of vitamin B_12_ acts synergistically with folate to precipitate NTDs on a sporadic basis. In other words, low vitamin B_12_ status (or a genetic predisposition based on vitamin B_12_‐related genetic variability) may confer an underlying risk that is manifested in an NTD‐affected pregnancy in combination with low folate status—but sufficiently low folate status on its own could also be sufficient to precipitate an event. This type of gene–nutrient interaction has been speculated in several studies related to NTDs, particularly in relation to the *MTHFR* 677C>T polymorphism.^55^, ^56^

### Vitamin B_12_–folate interaction in relation to NTD risk

One further point relevant to this argument relates to the known intracellular interaction of vitamin B_12_ with folate. As noted earlier, vitamin B_12_ deficiency results in an intracellular folate deficiency, due to an inability of cells to incorporate new folate molecules from the circulation and a trapping of existing intracellular folates as methyl folate. This is seen clinically as vitamin B_12_ deficiency being associated with lower RCF, whereas serum folate may be unchanged or even elevated. Examining this effect in the case of NTDs, Daly *et al*.^57^ and Crider *et al*.^58^ observed that risk of having an NTD‐affected pregnancy is strongly associated with maternal RCF and that improving RCF concentrations to beyond 906 nmol/L provides optimal protection against having an NTD‐affected pregnancy. Because of its interaction with folate, lower vitamin B_12_ status in women of reproductive age can act as a constraint toward achieving an RCF concentration that would be optimally protective against NTDs. This is borne out in the Kirke *et al*. study described above, where serum folate and vitamin B_12_ were not significantly associated, but the authors observed RCF to be positively associated with vitamin B_12_ in cases (*P* = 0.004) but not in controls (*P* = 0.73).^54^ The authors suggested that there may be a genetic predisposition linked to a folate and B_12_ interaction in women who had NTD‐affected pregnancies. This type of scenario was recently replicated in a population‐based study examining folate and vitamin B_12_ status in 937 women of reproductive age in Belize.^59^ RCF concentrations were examined after stratification by serum B_12_ concentration and other adjustment factors. Both folate and vitamin B_12_ deficiencies were widespread, and having vitamin B_12_ deficiency was shown to be an important obstacle to achieving optimal RCF concentrations.

To summarize, acknowledging that all the data presented above are observational and therefore must be considered low‐grade evidence, there is a strong argument to support the notion that low vitamin B_12_ status increases maternal risk for having an NTD‐affected pregnancy, independent of folate, and, in addition, that the known interaction between vitamin B_12_ and folate can create a synergism in relation to the magnitude of risk. This argument suggests that it would be wise to consider improving vitamin B_12_ status in conjunction with folic acid intervention to optimize the reduction of NTDs, although it is also possible that risk caused by low B_12_ could be overcome by supplementation with sufficient folic acid alone. To be specific, if the mechanism of folic acid protection against NTDs is primarily related to DNA synthesis and cell proliferation, folic acid, when converted to tetrahydrofolate (THF), could maintain a flow of one‐carbon units to purines and pyrimidines even in the situation of low vitamin B_12_ status, where methionine synthase is functional but not at an optimal level, and there is an increased flux of one‐carbon units to methyl folate (as noted earlier in the section on biological plausibility). Such a possibility does not suggest that very high doses of folic acid would be required but highlights the point that the concept of sufficient folic acid has not been addressed in any study and may vary on the basis of both nutrient and genetic factors.

## Is it likely that adding vitamin B_12_ in fortification programs would further reduce NTDs?

In addition to the comments raised in the previous section, there are other relevant data from the large prospective studies noted above that help to address the likely utility of adding vitamin B_12_ to fortification programs. There is a general consistency in the data from the studies noted in Table 3 in terms of the magnitude of risk conferred by low vitamin B_12_ status, in that none of the studies found a significant progression of risk reduction across the full range of B_12_ status quantiles. This contrasts with data on RCF status and risk of NTDs.^57^, ^58^ In all the reported vitamin B_12_–related studies, it appears that risk is constrained to blood concentrations that would be considered deficient or marginally deficient (i.e., concentrations below 200 pmol/L in the published studies). The observation is also supported by the absence of association of vitamin B_12_ with NTDs in the Finnish study in which maternal vitamin B_12_ status was high (mean > 350 pmol/L). Such an effect is biologically plausible and consistent with the known relationship of vitamin B_12_ status to metabolic function of B_12_‐dependent enzymes. The relationship is demonstrated by observations from several studies that serum vitamin B_12_ concentrations above approximately 250 pmol/L have no significant relationship with the vitamin B_12_ metabolic biomarker methylmalonic acid (MMA).^60^ In other words, once there is optimal activity of the two vitamin B_12_–dependent enzymes, adding additional vitamin B_12_ has no relevant biological function. In practice, the actual concentration of added vitamin B_12_ to achieve this goal may vary in different settings, such as the presence of malabsorption due to diverse factors^61^ and the effect of genetic factors that might impair the trafficking of B_12_ to its final destination.^62^, ^63^

It should be noted that, during pregnancy, the concentration of vitamin B_12_ in maternal blood undergoes a natural physiological drop to between 70% and 75% of nonpregnant levels by about 20 weeks gestation.^64^ The data noted above relate to a sample collection period in the region of 15–20 weeks gestation. To extrapolate the study findings to maternal prepregnancy status, the data imply that women entering pregnancy with vitamin B_12_ blood status below about 250 pmol/L are at highest risk, and there may be little additional benefit in improving B_12_ status beyond these values. This cutoff agrees very well with calculated cutoffs for the association of vitamin B_12_ status with MMA.^60^ Therefore, the question of whether it is likely that adding vitamin B_12_ in fortification programs would further reduce NTDs would have to be considered in the context of the prevalence of vitamin B_12_ deficiency (or low vitamin B_12_ status) in women of reproductive age across different populations and ethnic groups.

### Vitamin B_12_ deficiency

Severe clinical vitamin B_12_ deficiency is characteristically observed in individuals who have gastrointestinal disorders that affect their capacity to absorb B_12_ from all food sources, including oral supplements. Diagnosis is usually based on classical hematological or neurological symptoms, and the condition requires medical intervention.^65^ While the most common cause (pernicious anemia) can affect individuals of all ages, it is rare in women of reproductive age. The main contribution to the worldwide prevalence of vitamin B_12_ deficiency results from two factors: low intake of food sources that are rich in vitamin B_12_ (meat and dairy products) and infections with intestinal parasites.^12^, ^66^, ^67^, ^68^, ^69^, ^70^, ^71^

### Diagnosis issues

The diagnosis of vitamin B_12_ deficiency in population studies is generally not based on classical clinical symptoms but on low concentrations of serum total vitamin B_12_, typically defined as total serum B_12_ <148 pmol/L.^72^ Other blood biomarkers can also be used but are much less widely reported. These include holoTC, which measures vitamin B_12_ available for tissue uptake, plus plasma MMA and plasma total homocysteine, which measure functional or metabolic stress on the two vitamin B_12_–dependent enzymes.^72^, ^73^, ^74^, ^75^, ^76^, ^77^ While all four biomarkers have merit and are important guides to assessing population vitamin B_12_ status, all have limitations in sensitivity and specificity.^72^ Recently, a test based on a combination of these markers has received interest and may improve the diagnosis of deficiency,^78^ but it is not widely used. There are considerable financial and technical obstacles to setting up methodologies for the two functional biomarkers in situations of limited resources, and these are infrequently measured in population studies, so published estimates to date are based mainly on serum total vitamin B_12_, which is reasonably standardized across laboratories as a measure^72^ but is possibly the least reliable biological indicator, particularly when values fall into an indeterminate range that is neither adequate nor deficient,^79^ as discussed further below.

The prevalence of anemia in a population is an important additional aspect and is sometimes included in population studies of vitamin B_12_ and folate status, but iron deficiency is a common cause of anemia that often coincides with areas of low folate and/or vitamin B_12_ status,^67^, ^80^, ^81^, ^82^ such that using the presence of anemia to infer folate or vitamin B_12_ deficiency is unjustified. The prevalence of macrocytic anemia is generally regarded as a poor indicator of vitamin B_12_ status.^66^, ^83^ Because diagnosis beyond measurement of blood status is not performed, the presence of overt clinical symptoms due to vitamin B_12_ deficiency is not known. There is a considerable literature on what is termed *subclinical vitamin B_12_ deficiency*, where serum total vitamin B_12_ concentrations fall into a diagnostically uncertain range between 110 pmol/L and approximately 250 pmol/L, and objective clinical or metabolic features, such as anemia, are absent.^75^, ^84^ Subclinical deficiency is challenging to address either at a clinical or a public health level^85^ and is made even more uncertain in children and women of reproductive age, because different cutoff levels defining both subclinical and clinical cobalamin deficiency need to be used.^86^

The limitations noted above (i.e., biomarker insensitivity, lack of age‐ and physiology‐specific cutoffs) reflect a serious knowledge gap that needs to be addressed in order to properly understand the extent and urgency of the problem in relation to vitamin B_12_ deficiency and associated risk of NTDs (or other adverse pregnancy outcomes) in women of reproductive age. Collection of reliable data on vitamin B_12_ status, including data from intervention studies in different population groups and regions, is an important research goal. Notwithstanding these needs, in areas of high endemic anemia and a high prevalence of vitamin B_12_ concentrations lower than 250 pmol/L in women of reproductive age, indicating at least suboptimal function of vitamin B_12_–dependent enzymes, there is a strong case for immediate fortification of food or supplementation with vitamin B_12_. Such a strategy should be accompanied by collection of data on folate and B_12_ status to monitor the performance and effects of implementation.

### Global prevalence of vitamin B_12_ deficiency in women of reproductive age

Population and cohort or convenience‐based data indicate that there is a high prevalence of vitamin B_12_ deficiency (usually defined as serum total vitamin B_12_ < 148 pmol/L) among women of reproductive age in many parts of the world, and while this is not confined to low‐income countries,^87^, ^88^ such countries are at greatest risk of lifelong adverse consequences. The underlying reasons vary from unintended low intake of vitamin B_12_–rich food owing to poverty and limited access to deliberate exclusion of such food because of cultural or religious customs and to personal restraints (e.g., vegetarianism). A comprehensive review of the global prevalence of vitamin B_12_ deficiency is not intended here, but some examples are presented to give a sense of the problem to be addressed. For example, in South and Central America, national rates of deficiency plus marginal deficiency (i.e., serum vitamin B_12_ < 221 pmol/L) among women of reproductive age vary between 3% in Costa Rica, 8% in Mexico, 12% in Argentina, and 37% in Colombia.^89^ In Belize, the national prevalence of deficiency using the same upper cutoff was 50% in a recent study,^59^ and, in Venezuela, vitamin B_12_ deficiency was reported to be 11% nationwide.^90^ In India, Nepal, and Bhutan, the prevalence of vitamin B_12_ deficiency is reported to be extremely high^76^, ^91^, ^92^, ^93^, ^94^ and was more than 80% in one study of Indian adolescents in a low‐income area.^95^ It has been speculated that maternal vitamin B_12_ deficiency could be the most important risk factor for NTDs in vegetarian countries like India.^96^ In China, one recent population‐based study of 1170 women aged 10–49 years from Shanxi Province in Northern China reported a prevalence of 45.5% vitamin B_12_ deficiency (<148 pmol/L), and a further 25% of women had marginal deficiency (<220 pmol/L). Of these, approximately 10% had combined folate and vitamin B_12_ deficiencies.^68^

Studies on adolescents and women of reproductive age in Africa show that there is considerable divergence of vitamin B_12_ status across regions. This is likely due to genetic as well as nutritional factors. Deficiency is highly prevalent in some regions, and this seems to be associated with malnutrition and low consumption of animal‐source foods.^12^, ^70^, ^97^ For example, in Cameroon, 29% of women nationally had vitamin B_12_ concentrations < 221 pmol/L, but low status was most prevalent (40%) in poorer regions.^98^ By contrast, vitamin B_12_ deficiency does not appear to be a problem in other areas. For example, in a population study of 871 women of reproductive age in Sierra Leone, vitamin B_12_ status was high (mean (SD) of 556 (263) pmol/L), and the prevalence of deficiency (<150 pmol/L) was extremely low (<0.5%).^99^ In the Democratic Republic of the Congo, folate and vitamin B_12_ deficiencies were reported to be <5%,^100^ and in Ghana they were also negligible.^101^

### Influence of genetic factors on vitamin B_12_ status

The apparent conundrums in relation to the prevalence of vitamin B_12_ deficiency across different populations might be explained to some extent by genetic differences between racial and ethnic groups. Studies both from the African continent and the United States consistently demonstrate significantly higher concentrations of total vitamin B_12_ and the two serum B_12_‐binding proteins TCII and haptocorrin in black compared with white subjects.^102^, ^103^, ^104^, ^105^, ^106^, ^107^ These racial differences are observed across the life span, from cord blood samples in newborn infants to healthy elderly. Data from the NHANES study also suggest that Mexican American populations have higher serum vitamin B_12_ concentrations than non‐Hispanic whites, but not as high as non‐Hispanic blacks. For example, compared with non‐Hispanic whites, Mexican Americans and non‐Hispanic blacks have serum B_12_ concentrations that are 10% and 21% higher, respectively.^106^ It is unlikely that the differences between black and white populations are environmental, and therefore genetic factors are likely to be involved.^104^, ^106^, ^107^

The genetic architecture of serum vitamin B_12_ in humans has been explored in several genome‐wide association studies. As expected, known genes involved in vitamin B_12_ trafficking from dietary intake to cellular uptake are involved,^62^, ^63^ but the strongest signal determining serum B_12_ status is the fucosyltransferase gene (*FUT2*). This controls the secretion of ABH blood group antigens into body fluids, such as saliva and gastrointestinal fluids, and may be an important modifier of disease and infection.^62^, ^108^, ^109^, ^110^ It is not clear whether the relationship of *FUT2* with serum vitamin B_12_ is due to microbiome interactions or other factors. One study examined the relationship between polymorphisms in *FUT2* and helicobacter infection, a known cause of lower vitamin B_12_ status, but there was no significant association.^107^ Such gene–nutrient–infection interactions remain poorly explored. The main point here is that, while differences due to genetic variability may be small in magnitude, it is not known how subtle alterations in vitamin B_12_ status might change the equilibrium that exists between the human host and gastrointestinal parasites or how much vitamin B_12_ should be included in a fortification program to optimize improvement in status and to ensure that no adverse interaction with infectious parasites is promoted. This area constitutes a major research need.

Genetic variability across different racial groups is known to be an important element determining the risk for NTDs. The role of genes in determining micronutrient status could be an important factor in determining the risk for NTDs, but this is a hypothesis, not a proven fact. For example, the well‐described *MTHFR* 677C > T polymorphism, noted earlier in relation to NTD risk, is a cause of low serum and RCFs.^111^, ^112^, ^113^ This polymorphism has a wide variation in prevalence worldwide, with the rare TT genotype being extremely low in African populations studied to date, moderately prevalent (5–15%) in Caucasian populations, and high in Hispanics and some Chinese ethnic groups.^114^, ^115^, ^116^, ^117^ Of interest, but not biologically understood, is the paradox in the United States of non‐Hispanic black women who have the highest vitamin B_12_ status and a 10‐ to 20‐fold lower prevalence of the *MTHFR* TT genotype than other measured racial groups^113^ but also have the lowest risk for an NTD‐affected pregnancy among U.S. population groups, in the face of also having the lowest folate and RCF status among women of reproductive age.^118^, ^119^, ^120^ Of further interest is the observation of the least change in prevalence of NTDs among non‐Hispanic black women following mandatory folic acid fortification.^120^ One might speculate that low folate status is of less consequence if vitamin B_12_ status is adequate, but such an interpretation seems to contradict data discussed earlier suggesting that low folate status per se is sufficient to cause increased maternal risk of having an NTD‐affected pregnancy. These conundrums highlight the fact that interactions between genetic and nutritional factors in relation to risk of NTDs are highly complex and poorly understood. It is highly likely that underlying racial/ethnic predisposition to NTD occurrence and/or to low vitamin B_12_ status will be important factors in establishing the benefit of adding vitamin B_12_ to a fortification program.

## Research issues in relation to vitamin B_12_ in fortification programs

### Research gaps in relation to need

Concern has been raised in regard to supplementing women of reproductive age in low vitamin B_12_–intake areas with folic acid but not vitamin B_12_. This is an active research area, and the question of whether supplementation with vitamin B_12_ can counteract apparent adverse metabolic effects of high‐dose folic acid supplementation in mothers or offspring (such as insulin resistance/“diabesity”)^121^, ^122^, ^123^ is currently being addressed, although much more research is needed. However, as briefly outlined earlier, there are large variations in the extent and even in the apparent susceptibility to vitamin B_12_ deficiency between populations. Therefore, while targeted programs of fortification or supplementation should be implemented in areas of high endemic need, a general global strategy of recommending fortification with vitamin B_12_ along with folic acid could be premature or perhaps purposeless in advance of more research. There is an argument that it will do no harm and will at least address potential adverse effects of excessive folic acid intakes. This argument is also speculation in the absence of more research on appropriate dosage or on potential interactions of oral vitamin B_12_ with the human microbiome and with parasitic or infectious agents, as noted below.^124^, ^125^, ^126^

### Research gaps in relation to dosage

The gastrointestinal process leading to the absorption of vitamin B_12_ is intricate and culminates in vitamin B_12_ (linked to intrinsic factor) being absorbed through specific B_12_ receptors in the terminal ileum. Under physiological conditions, these receptors become saturated at between 1.5 and 2 μg per dose.^127^ There is good evidence from intervention studies that an intake of 4–6 μg/day is adequate to achieve a normal status in healthy individuals,^128^ and, interestingly, the amount of vitamin B_12_ in the supplement given to women in the Hungarian trial was 4 μg/day.^41^ However, a recent evaluation of the existing Palestinian vitamin B_12_ fortification program suggests that the very small amounts currently added to food products aiming to achieve an intake of 4 μg/day may not be sufficient to eliminate B_12_ deficiency where consumption of animal‐derived proteins is extremely low.^129^ The proportion of oral B_12_ that is absorbed drops dramatically as the dose is increased, such that, for example, about 15% of a 10 μg dose and less than 1% of a 25–50 μg dose is absorbed.^12^, ^127^, ^128^, ^130^ The fraction of vitamin B_12_ absorbed passively is limited to about 1–2% of the dose. These data suggest that there is little value in giving large doses to persons with healthy gastrointestinal function. A variety of intervention studies have examined the effect of daily vitamin B_12_ supplements from 2.5 to 1000 μg on blood vitamin B_12_ status and other health outcomes.^92^, ^122^, ^130^, ^131^, ^132^, ^133^, ^134^ Several publications have also examined cofortification with low doses of vitamin B_12_ (10–20 μg) and folic acid^49^, ^135^ in relation to improvement in dietary status. Some of these have shown efficacy in increasing the serum vitamin B_12_ concentration of participants but not necessarily improvements in medical outcomes being measured.^92^, ^133^, ^136^ In an analysis of the optimal amount of vitamin B_12_ to add in a fortification program, based on available evidence, an intake of 20 μg/day was recently recommended.^135^ This seems like a reasonable balance between ensuring some effect and preventing unnecessary excess. Nevertheless, given NTD prevention as a primary outcome in relation to vitamin B_12_ status, intervention studies in women of reproductive age remain an important research target. In relation to this, some recent vitamin B_12_ intervention studies have been carried out on pregnant and lactating women, using B_12_ supplements up to 50 μg/day.^137^, ^138^ These have demonstrated increased vitamin B_12_ status of mothers and infants, but the specific goal of ensuring adequate B_12_ status for women entering pregnancy has not been specifically addressed.

### Research gaps in relation to toxicity

To date, there is no known toxicity of vitamin B_12_.^139^ Long‐term intake (either oral or parenteral) of high doses of the vitamin, as documented in patients with pernicious anemia,^140^ has not been associated with adverse effects, and vitamin B_12_ has not been reported to be teratogenic, carcinogenic, or toxic. However, as noted earlier, a large fraction of a vitamin B_12_ dose is not absorbed and will be available to the human microbiome for further metabolism to biologically inactive compounds^141^ or to parasites that consume the vitamin.^142^ More research is needed on potential interactions between vitamin B_12_, the human microbiome, and parasitic infections of the gastrointestinal tract to confirm that no unexpected negative consequences emerge from altering the balance between host and parasite vitamin B_12_ status and to establish a beneficial regimen of fortification in areas where such diseases are endemic.

## Summary

In relation to reducing the risk of NTDs, the present state of scientific knowledge does not support food fortification with vitamin B_12_ with the same degree of certainty as that for folic acid, although the existing evidence strongly suggests a relationship between low maternal vitamin B_12_ status and increased risk of NTDs. Nevertheless, the known synergy between vitamin B_12_ and folate makes it highly probable that fortification with vitamin B_12_ will improve the efficacy of current folic acid fortification programs in the prevention of NTDs. The case is much stronger in parts of the world where low and deficient vitamin B_12_ status is endemic in women of reproductive age, but, even in high‐income countries, the benefit of low‐dose vitamin B_12_ fortification probably outweighs any speculated negative consequences,^126^ although more research is needed to establish the efficacy of low‐dose fortification, the potential interactions of vitamin B_12_ with microbiome and parasitic agents, and the influence of genetic variability on vitamin B_12_ status. In countries with a high prevalence of B_12_ deficiency, consideration of B_12_ inclusion with folic acid in fortification programs should be a top priority.

## Competing interests

The author declares no competing interests.
